# Characteristics of piRNAs and their comparative profiling in testes of sheep with different fertility

**DOI:** 10.3389/fgene.2022.1078049

**Published:** 2022-12-07

**Authors:** Ran Di, Rensen Zhang, Joram Mwashigadi Mwacharo, Xiangyu Wang, Xiaoyun He, Yufang Liu, Jinlong Zhang, Yiming Gong, Xiaosheng Zhang, Mingxing Chu

**Affiliations:** ^1^ Key Laboratory of Animal Genetics, Breeding and Reproduction of Ministry of Agriculture and Rural Affairs, Institute of Animal Science, Chinese Academy of Agricultural Sciences, Beijing, China; ^2^ School of Advanced Agricultural Sciences, Yiyang Vocational & Technical College, Yiyang, China; ^3^ Small Ruminant Genomics International Center for Agricultural Research in the Dry Areas (ICARDA), Addis Ababa, Ethiopia; ^4^ Institute of Animal and Veterinary Sciences, SRUC and Center for Tropical Livestock Genetics and Health (CTLGH), Midlothian, United Kingdom; ^5^ Institute of Animal Husbandry and Veterinary Medicine, Tianjin Academy of Agricultural Sciences, Tianjin, China

**Keywords:** piRNAs, sheep, testis, reproduction, ping-pong signature

## Abstract

As a novel class of small RNAs, piRNAs are highly expressed in the animal gonads and their main known role is to inhibit transposon activity for ensuring the correctness and integrity of genome. In order to explore the characteristics of piRNAs in sheep testis and their possible regulatory roles on male reproduction, deep sequencing technology was used to sequence small RNAs and identify piRNAs in testes of sheep. The length of piRNAs in sheep testes showed a unimodal distribution between 26 and 31 nt, with a peak at 29 nt. These piRNAs exhibited obvious ping-pong signature and strand specificity. In the genome, they were mainly aligned to CDS, intron, repetitive sequence regions and unannotated regions. Furthermore, in transposon analysis, piRNAs were aligned predominantly to LINE, SINE, and LTR types of retrotransposon in sheep testes, and the piRNAs derived from each type showed obvious ping-pong signature. The piRNA clusters identified in sheep testes were mainly distributed on chromosomes 3, 7, 15, 17, 18 and 20. The results combining semen determination with pathway enrichment analysis implied that differentially expressed piRNAs between the testes of rams with different fertility might participate in spermatogenesis by regulating multiple pathways closely related to stabilization of blood-testis barrier and renewal and differentiation of spermatogonial stem cell. Taken together, the study provided new insights into the characteristics, origin and expression patterns of piRNAs in sheep testes tissue, which would help us better understand the role of piRNAs in sheep reproduction.

## 1 Introduction

piRNAs are a novel class of small RNAs different from microRNAs. They were firstly discovered in the testes tissue of *Drosophila melanogaster* (Aravin et al., 2001). They are far more numerous than microRNAs and are distinctly different from microRNAs in length, sequence characteristics and distribution. Usually, the lengths of piRNAs are between 24 and 33 nt (Czech et al., 2018; Ozata et al., 2019). They must specifically bind PIWI proteins to exert their biological functions, so they are called PIWI-interacting RNA (piRNA) (Aravin et al., 2006; Girard et al., 2006; Houwing et al., 2007). piRNAs include two forms: primary piRNAs and secondary piRNAs (Czech et al., 2018; Ozata et al., 2019). Primary piRNAs are mainly processed from precursor piRNAs which were transcript from piRNAs clusters, and usually their 5′ ends are base U (Vagin et al., 2006; Houwing et al., 2007). Then primary piRNAs bind with a kind of PIWI protein (Aub in *Drosophila*, Miwi in mouse) to form a complex, and they together recognize and cleave RNAs transcribed from transposon. Thus, secondary piRNAs with A preference at 10th base are formed. The secondary piRNAs can combine with another PIWI protein (Ago3 in *Drosophila*, Mili in mouse) to form a complex that is just enough to cleave the precursor piRNAs and generate primary piRNAs. Through this ping-pong cycle mechanism, the function of piRNAs to inhibit transposon transcription is realized (Brennecke et al., 2007; Gunawardane et al., 2007; Aravin et al., 2008; Toth et al., 2016; Madison-Villar et al., 2017).

Although the biological origin and function of piRNAs have not been fully elucidated, more and more evidence suggested that piRNAs are critical for reproduction. piRNAs are highly expressed in animal gonads (Grivna et al., 2006; Vagin et al., 2006; Houwing et al., 2007), and play important roles in maintaining integrity of the gamete genome (Vagin et al., 2006; Goriaux et al., 2014; Mohn et al., 2014; Ernst et al., 2017), gender determination (Kiuchi et al., 2014), germ cell differentiation (Gonzalez et al., 2015) and epigenetic regulation of gene expression (Teixeira et al., 2017; Czech et al., 2018). During spermatogenesis in mice, piRNAs are involved in the degradation of multiple mRNAs, whose expression is detrimental to spermatogenesis (Watanabe et al., 2011; Gou et al., 2014). Multiple studies suggested that piRNAs are essential in animal spermatogenesis (Goh et al., 2015; Zhang et al., 2015; Guo et al., 2018). Recently, we found that PIWI protein had the highest expression level in the gonad of sheep compared with other tissues (Li et al., 2020). Furthermore, the expression in testes is higher than that in ovaries (Li et al., 2020), which implies the important roles of piRNAs in male gonad.

The sequence information and visual map of piRNAs in humans and some model organisms (mouse, rat, *Drosophila*) can be accessed from a piRNA bank database (http://pirnabank.ibab.ac.in/) (Lakshmi and Agrawal, 2008). Recently, the database piRBase (http://bigdata.ibp.ac.cn/piRBase) collected piRNA sequence information from 44 species, and this information included gold standard piRNA sets, piRNAs expression data, regulatory relationship between piRNAs and target genes, and so on (Wang et al., 2022). However, the information about ovine piRNAs sequences can’t retrieved from these two databases. In 2019, He et al. (2019) predicted piRNAs in sheep testes tissue. However, the in-depth sequence features (including ping-pong signature, strand specificity, the genomic distribution and other characteristics of sheep piRNAs) still need to be fully revealed. Therefore in this study, solexa sequencing technology and bioinformatics methods were used to screen and identify piRNA sequences in sheep testes, and their sequence characteristics, genomic origin and chromosomal distribution were revealed. Then, differentially expressed (DE) piRNAs in the testes of rams with different fertility were analyzed and their functions were predicted by combining the results of target gene function clustering and semen index assay. These would help us better understand sequence characteristics and origin of sheep piRNAs and provide new insights into the epigenetic regulation of ram fertility.

## 2 Materials and methods

### 2.1 Sheep semen evaluation and testicular tissue collection

In china, both Small-Tailed Han (STH) sheep and Sunite (SNT) sheep belong to the Chinese Mongolian sheep clade and have similar genetic backgrounds. However, their fertilities were distinct (STH with high fertility and SNT with low fertility). In this study, we selected a sheep farm where both breeds were raised in Tianjin of China, and the feed and management were the same for both breeds in this farm. Then six healthy, 3-year-old adult rams with similar weight (difference less than 1 kg) were selected in two breeds (3 rams in each breed) for semen evaluation and sample collection. At AM 9:00, semen was collected when the ram was fully climbing the back of the ewe model in order to ensure maximum semen volume in each collection. For all the rams, semen was collected three times simultaneously within 10 days. Ejaculate volume, sperm motility, viable sperm ratio, and sperm density were determined using the methods described by David et al. (2015). The pH of fresh essence was measured with a SevenExcellence™ pH meter. The semen slides were stained with 0.5% gentian violet, and 500 sperms in different view fields were viewed under a microscope to calculate the percentage of abnormal sperm. The above data were analyzed by one-way ANOVA with SPSS19.0, and *p* < 0.05 was considered to be statistically significant. After all measurements were completed, the above six rams were euthanized at the same time, and the testes of the rams were immediately collected and quickly stored in liquid nitrogen.

### 2.2 RNA extraction and small RNA sequencing

Total RNA was extracted from the testes according to the instructions of the TRIzol kit. The Agilent 2100 Bioanalyzer was used to accurately detect RNA integrity, and the RIN value of each sample was greater than 8.5 which indicates good quality of RNA for subsequent analysis. Small RNAs less than 40 nt in length were isolated from total RNA using a FlashPAGE fractiontor (Ambion, Life Technologies, Paisley, UK), and adapters (5′ seq: 5′-GUU​CAG​AGU​UCU​ACA​GUC​CGA​CGA​UC-3’; 3′ seq: 5′-TGG​AAT​TCT​CGG​GTG​CCA​AGG-3′) were attached to both ends of the isolated small RNAs using the Illumina TruSeq Small RNA Sample Preparation kit. The cDNA synthesis was performed by RT-PCR and PCR products were screened using 15% polyacrylamide gels. RNA fragments with a length of 140–160 bp (the non-coding small RNA plus the 3′ and 5′ end adapter sequences) were recovered and sequenced using Illumina 2000. The construction and sequencing of the small RNA libraries were completed by Beijing Nuohezhiyuan Technology Co., Ltd. The raw sequencing data have been deposited in the Genome Sequence Archive (Chen et al., 2021) in National Genomics Data Center (Xue et al., 2022), China National Center for Bioinformation/Beijing Institute of Genomics, Chinese Academy of Sciences (GSA: CRA008325) that are publicly accessible at https://ngdc.cncb.ac.cn/gsa.

### 2.3 PIWI-interacting RNA identification

The obtained small RNA raw data was filtered to remove the following reads: low-quality reads (reads with Qphred≤5 bases accounting for more than 50% of the entire read length), reads with N bases content greater than 10%; reads containing 5′ adapter contamination, reads without 3′ adapter sequences and inserts; reads containing poly A/T/C/G (they may be due to sequencing errors). Then the 3′ linker sequence of the remaining data was removed, and finally clean reads were obtained after removal of atypical length range (<24 nt or >33 nt) reads of piRNA (Czech et al., 2018; Ozata et al., 2019). Furthermore, the clean reads from known other types of small RNAs (rRNAs, tRNAs, snRNAs, snoRNAs, and miRNAs) were removed by aligning them to the Rfam database (http://rfam.xfam.org/) (Kalvari et al., 2018) and miRbase v21.0 (http://www.mirbase.org/) (Griffiths-Jones et al., 2008). Then the new predicted miRNA reads by Mireap 0.2 were also removed from the remaining reads. Finally, reads with 1U or 10 A feature in the remaining reads were predicted as candidate piRNAs reads. Among them, piRNAs reads with the same sequence were considered as a piRNA tag.

### 2.4 Characterization of PIWI-interacting RNA sequences in sheep testes

First, the length distribution of piRNA reads and tags (reads with the same nucleotide sequence as a tag) in sheep testes tissue were counted, and the base frequency of each site in the 5′-3′ direction was analyzed. The putative piRNA sequences were then aligned to the sheep genome (Oar v 4.0) and the genomic composition of piRNAs was determined by bowtie2 (version 2.3.5). In the repeat region, piRNA is mainly derived from the transposable element (TE), therefore the piRNAs derived from the repeat regions were aligned with the TE sequences in Rpbase, thus the types of piRNAs in the TE were determined by RepeatMasker (version 4.1.0). The ping-pong structural feature means that there is a 10-base complementarity between the primary and secondary piRNA strands. We used the method described previously (Yan et al., 2011) to analyze the ping-pong structure of all putative piRNA sequences as a whole and piRNA sequences derived from each TE type in sheep testes, respectively.

### 2.5 Identification and analysis of PIWI-interacting RNA clusters in sheep testes

Most piRNAs are located in piRNA clusters scattered throughout the genome. Therefore, proTRAC v2.1.2 (Rosenkranz and Zischler, 2012; Rosenkranz, 2016) was used to identity piRNA clusters in the sheep genome. Then, strand specificity within individual piRNA cluster and all clusters was analyzed. The percentage of U bases in the first bases of all piRNAs derived from individual piRNA cluster were counted respectively. In addition, the distribution of piRNA clusters across chromosomes of sheep was also analyzed.

### 2.6 Screening and function prediction of differentially expressed PIWI-interacting RNAs in testes of sheep with different fertility

The piRNA expression levels of each sample were normalized using TPM. The DE piRNAs were screened between testes of sheep with high fertility and low fertility by DESeq2 program (v 1.4.5). Those piRNAs with *p*-value <0.05 and fold change >1.5 were considered to be differentially expressed between the two groups. The programs miRanda, RNAhybrid and TargetScan were used to predict target genes of DE piRNAs, and the overlap genes among prediction results of the three programs were screened as the candidate target genes for further analysis. DAVID software (https://david.ncifcrf.gov/) (Huang et al., 2009b; Huang et al., 2009a) was used for GO and KEGG enrichment analysis of target genes. Associations between the pathways of interest were analyzed using the emapplot function in the R cluster Profiler package. In addition, the interaction network among DE genes in male reproduction-related pathways was constructed using the enrichplot function in the R cluster Profiler package.

### 2.7 Quantitative polymerase chain reaction for sequencing validation

Quantitative polymerase chain reaction (qPCR) was employed to verify the accuracy of small RNA sequencing. U6 was used as a reference gene, and five piRNAs were randomly selected. The stem-loop primer method was used to improve the specificity of detection. Reverse transcription stem-loop primers and qPCR amplification primers were designed and showed in Supplementary Table S1. Total RNA was reverse transcribed to synthesize cDNA using PrimeScript™ RT Reagent Kit (Takara, Japan). The qPCR reaction conditions were as follows: 95°C for 15 min, followed by 40 cycles of 10 s at 95°C and 30 s at 60°C, respectively. Three technical replicates were performed for each sample, and the relative expression ratios of the piRNAs were calculated using the 2^−ΔΔCt^ method (Livak and Schmittgen, 2001), and processed by one-way ANOVA with SPSS 19.0.

## 3 Results

### 3.1 Detection of semen in rams

Sheep semen quality is an important factor affecting sheep fertility. Therefore, in order to explore whether there were significant differences in semen quality between SNT and STH rams, the volume of single ejaculate, sperm density, sperm number, pH value of semen, sperm motility, viable sperm ratio, and sperm deformity rate were detected respectively. The statistical results showed that the volume of single ejaculate, sperm number and pH value of semen in STH sheep were significantly higher than those of semen in SNT sheep (Table 1). For other parameters (including sperm motility, viable sperm ratio, sperm density and sperm deformity rate), there was no significant difference between the two breeds.

**TABLE 1:** Results of semen and sperm assay in SNT and STH rams.

Breeds	Ejaculate volume (ml)	Sperm density (100 million/ml)	Sperm number (100 million)	pH	Sperm motility	Viable sperm ratio	Sperm deformity rate
SNT	1.10 ± 0.22^B^	37.48 ± 5.51	40.68 ± 7.66^B^	5.85 ± 0.31^B^	5.00 ± 0	0.88 ± 0.03	0.15 ± 0.03
STH	1.66 ± 0.20^A^	35.69 ± 3.64	58.93 ± 7.86^A^	6.32 ± 0.21^A^	5.00 ± 0	0.86 ± 0.03	0.14 ± 0.02

Different letters (A, B) in superscript indicates significant differences (*p* < 0.01).

### 3.2 Identification of PIWI-interacting RNA sequences in sheep testes tissue

In order to reveal the sequence characteristics of piRNAs in sheep testes tissue, the small RNAs in sheep testes were firstly sequenced using solexa sequencing technology. A total of 44,388,241 and 46,551,224 small RNA raw reads were acquired in SNT and STH testes, respectively. After filtering low quality reads, we obtained 43,554,297 and 40,476,683 clean reads in SNT and STH testes (Table 2). Among them, more than 80% small RNAs can be aligned to the sheep genome. Finally, after removing the following sequences (including rRNA, tRNA, snRNA, snoRNA, known and newly predicted miRNA sequences) from these mapped reads, we obtained 4218952–6894,453 piRNAs reads and 775532–837165 piRNAs tags in testis of each ram for further analysis.

**TABLE 2:** Summary of small RNA sequencing data in sheep testes.

Items	SNT_1	SNT_2	SNT_3	STH_1	STH_2	STH_3
Total reads	13270313	13711506	17406422	15033708	15782638	15734878
N% > 10%	615	915	1162	1038	1034	1072
Low quality	54247	24537	30069	29023	28626	29159
5′ adapter contamine	6166	5279	11896	6654	9111	6548
3′ adapter null or insert null	262996	105401	245857	119728	151666	128713
With ployA/T/G/C	58340	50154	65763	76923	81184	70152
Clean reads	12887949	13525220	17051675	14800342	15511017	15499234
Mapped sRNA	10370445	11298862	14473027	12558212	12933495	13132236
Known_miRNA	968071	1136244	2442048	461349	560774	656669
rRNA	216009	258964	274155	166173	242957	172013
tRNA	8	7	7	4	6	3
snRNA	14138	18189	22600	16043	21977	16273
snoRNA	51218	39628	51331	21637	21177	24833
Novel_miRNA	364	1199	680	188	181	282
piRNA reads	4218952	4403608	4761580	5451634	6894453	6781895
piRNA tags	829876	794496	837165	775532	801650	800046

### 3.3 Sequence characteristics of sheep testis-derived PIWI-interacting RNAs

**FIGURE 1:**
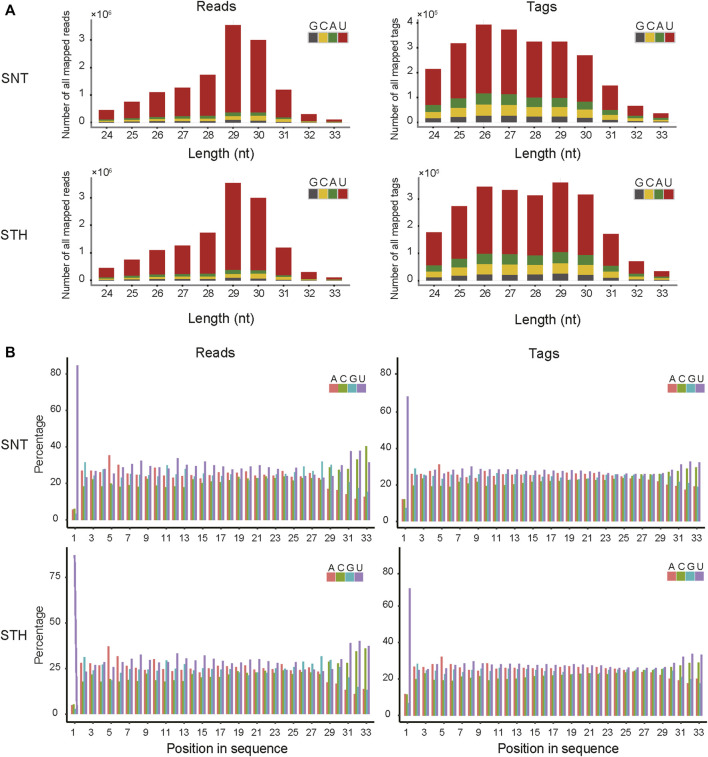
The feature of length and base composition of piRNAs in sheep testis. **(A)** Length distribution feature and base composition of the first nucleotide at 5′ end; **(B)** 5′-3′ base distribution frequencies of piRNAs reads and tags.

**FIGURE 2:**
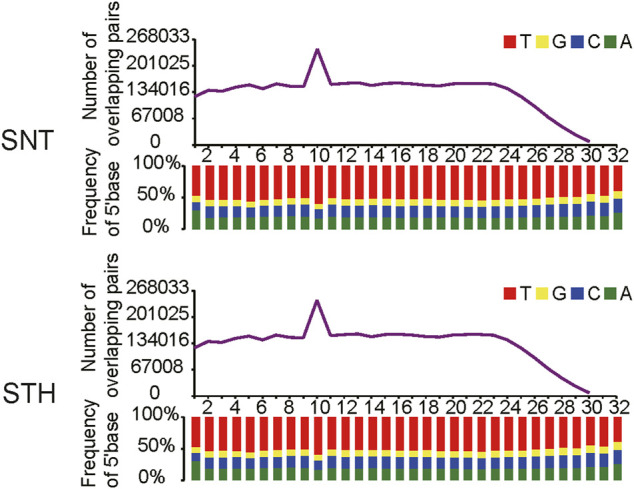
Ping-pong signature of piRNAs in sheep testis.

First, the length distribution of piRNAs reads and tags were analyzed in sheep testes tissue. The results in Figure 1A showed that the reads of piRNAs in sheep testes exhibit a unimodal distribution between 24 nt and 33 nt, with a peak at 29 nt. Most of piRNAs tags were concentrated between 25 nt and 30 nt. Simultaneously, we performed statistics on the base distribution frequencies of piRNAs reads and tags in the sheep testes. Their first bases in sequences had strong U preference (Figure 1), whereas the 10th base did not have base preference, implying that piRNAs in sheep testes mainly came from the primary piRNA pathway. In addition, the sequences of piRNAs in sheep testes exhibited strong ping-pong signature (Figure 2).

### 3.4 Genomic origin of sheep testes PIWI-interacting RNAs

**FIGURE 3:**
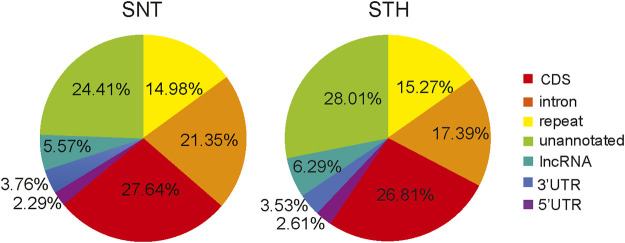
Genome distribution of sheep testis-derived piRNAs.

The piRNAs derived from different genomic regions play different biological roles. Therefore, in this study, the candidate piRNAs were aligned to the sheep genome for determining the main sources of piRNAs and their functional regions on the genome. Results showed that the alignment regions of piRNAs were similar in the testes of SNT and STH sheep, and they were mainly aligned to the CDS region, intron region, repetitive sequence region and unannotated region (Figure 3). In addition, a small part of piRNAs were aligned to 5′UTR, 3′UTR and lncRNA. All these regions can be considered as potential target regions regulated by piRNAs in sheep testis.

**FIGURE 4:**
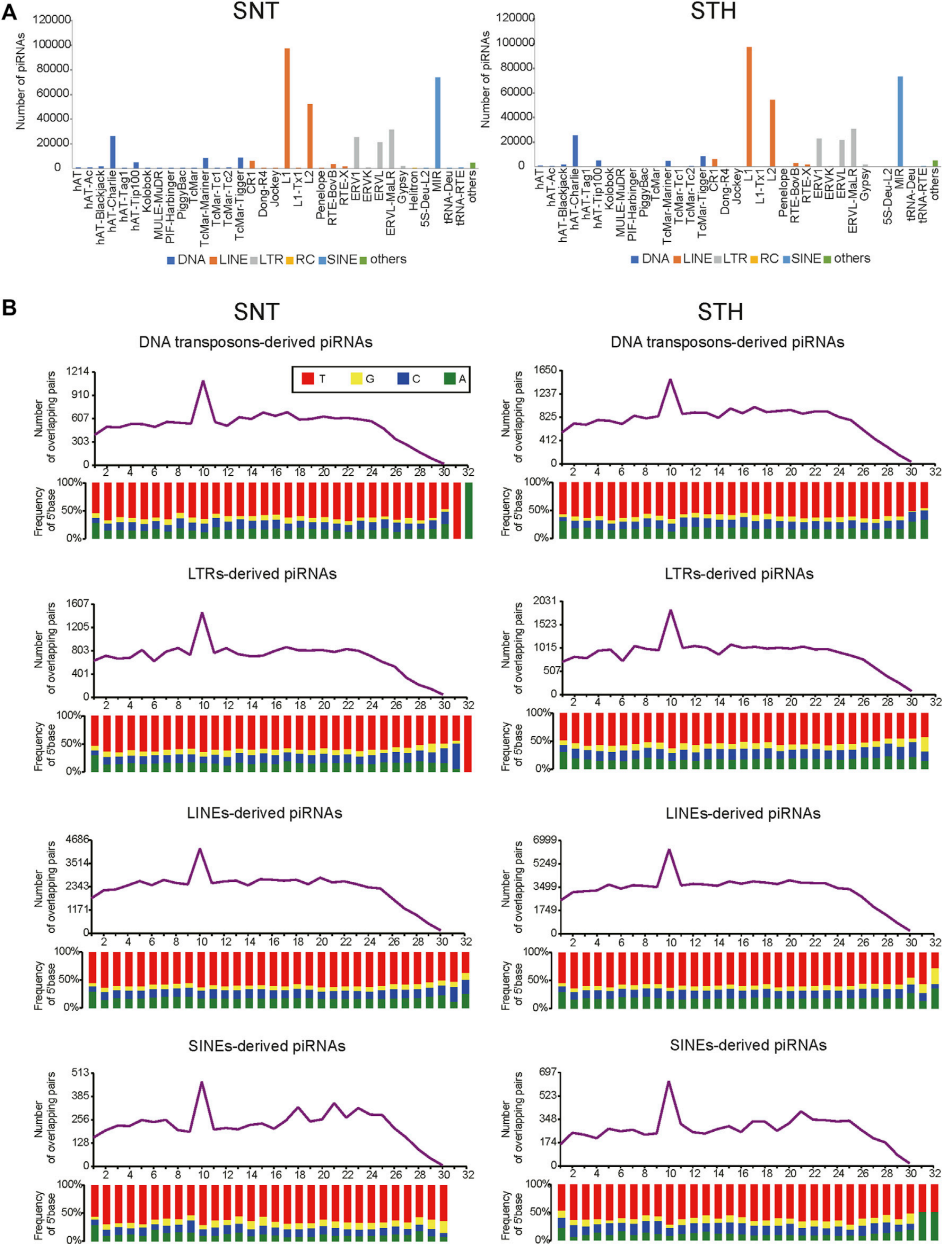
Distribution of sheep testis piRNAs among transposon elements **(A)** and the ping-pong signature of transposable element derived piRNAs in sheep testes **(B)**.

One main function of piRNA is to inhibit the activity of transposons. Therefore in this study, piRNAs derived from repeat sequences in sheep testes were aligned to the Repbase database in order to identify the types of transposable elements which piRNAs originate from and act in. Results showed that piRNAs in sheep testes were aligned predominantly to retrotransposon. The types of transposons were similar for piRNAs in SNT and STH testes, and they mainly included LINE, SINE, and LTR types in retrotransposons (Figure 4A). In addition, some piRNAs were also aligned to DNA-type transposons. In a finer classification, piRNAs were mainly aligned to L1 and L2 types in LINE, MIR type in SINE, ERV1, ERVK, ERVL, ERVL-MaLR types in LTR, and hAT-Charlie in DNA-type transposons. Furthermore, the piRNAs derived from each of the above TE types presented obvious ping-pong signature in sheep testes (Figure 4B), confirming that the piRNAs derived from each TE type can inhibit transposon activity through ping-pong cycle mechanism.

### 3.5 Characteristics and chromosomal distribution of PIWI-interacting RNA clusters

**FIGURE 5:**
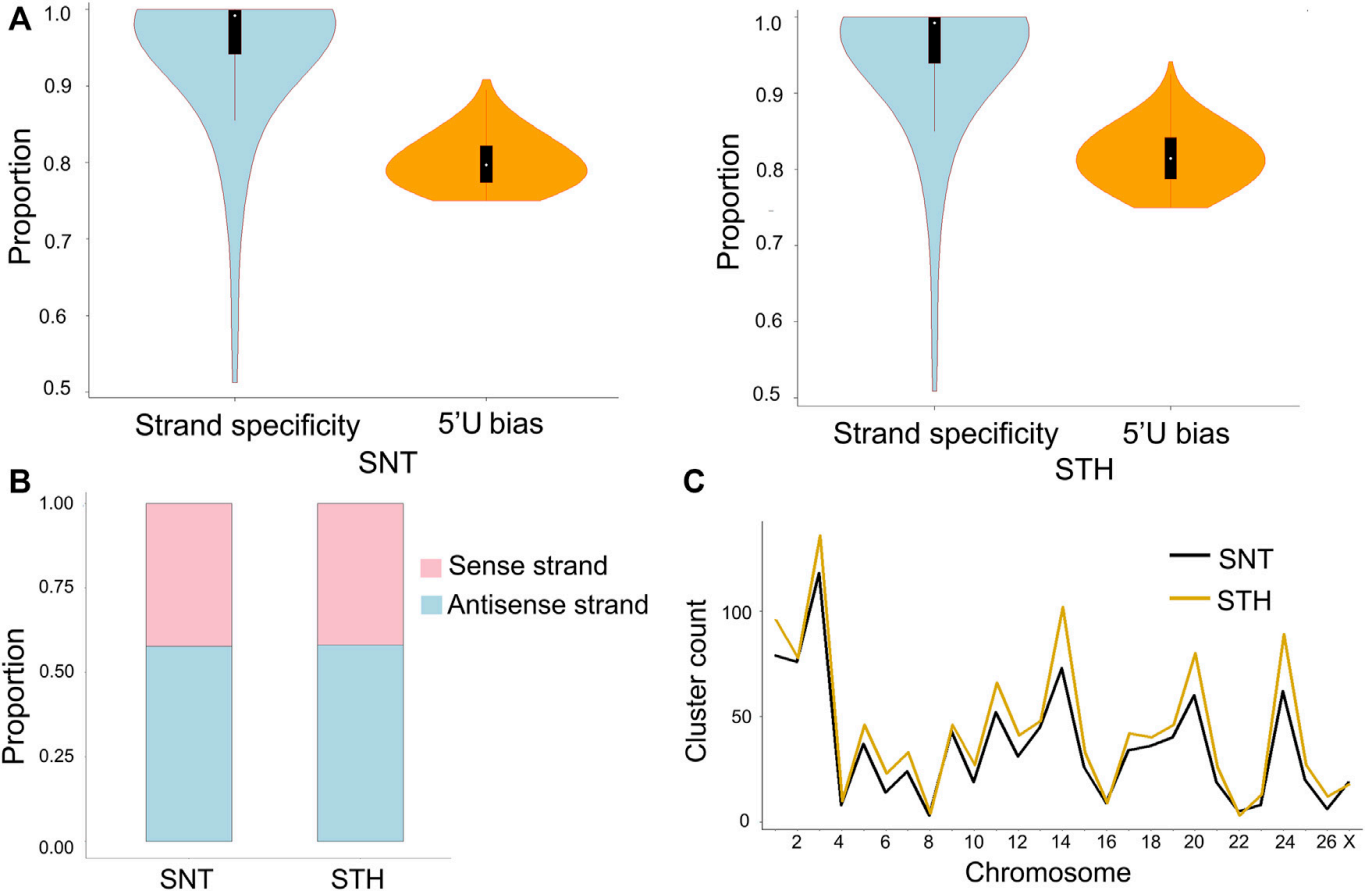
Characteristics of piRNA clusters in sheep testes. **(A)** Strand specificity and 5′U bias of piRNAs in clusters; **(B)** piRNAs distribution on the sense and antisense strands; **(C)** Chromosome distribution of piRNA clusters in testes of SNT and STH.

In most species, piRNAs exist in piRNAs clusters on genome. In testis of each ram, a total of 284–430 piRNA clusters were identified on genome. In each piRNA cluster, piRNAs had strong strand specificity in sheep testes (Figure 5A). However when all piRNA clusters were taken into account, the ratio of piRNAs derived from the sense and antisense strands was comparable (42% and 58%), and there was no longer obvious strand specificity (Figure 5B). At the same time, the first bases of all piRNAs derived from piRNA clusters had a strong U preference, which means that most piRNAs in clusters originated from the primary piRNA pathway. In addition, results showed that the distribution of piRNA clusters was not proportional to the length of chromosomes. In sheep testes, piRNA clusters were mainly distributed on chromosomes 3, 14, 20, and 24 (Figure 5C), which implied that piRNA clusters on these chromosomes have more important roles in maintaining the stability of germ cell genome and regulating the physiological activity of testis in rams.

### 3.6 Screening of differentially expressed PIWI-interacting RNAs in testes of rams and their target gene function prediction

**FIGURE 6:**
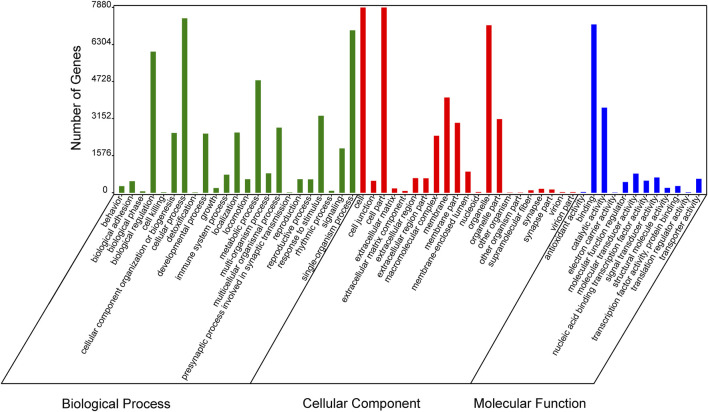
Enriched Gene Ontology (GO) terms of DE-piRNAs targeted gene between the testes of SNT and STH sheep.

**FIGURE 7:**
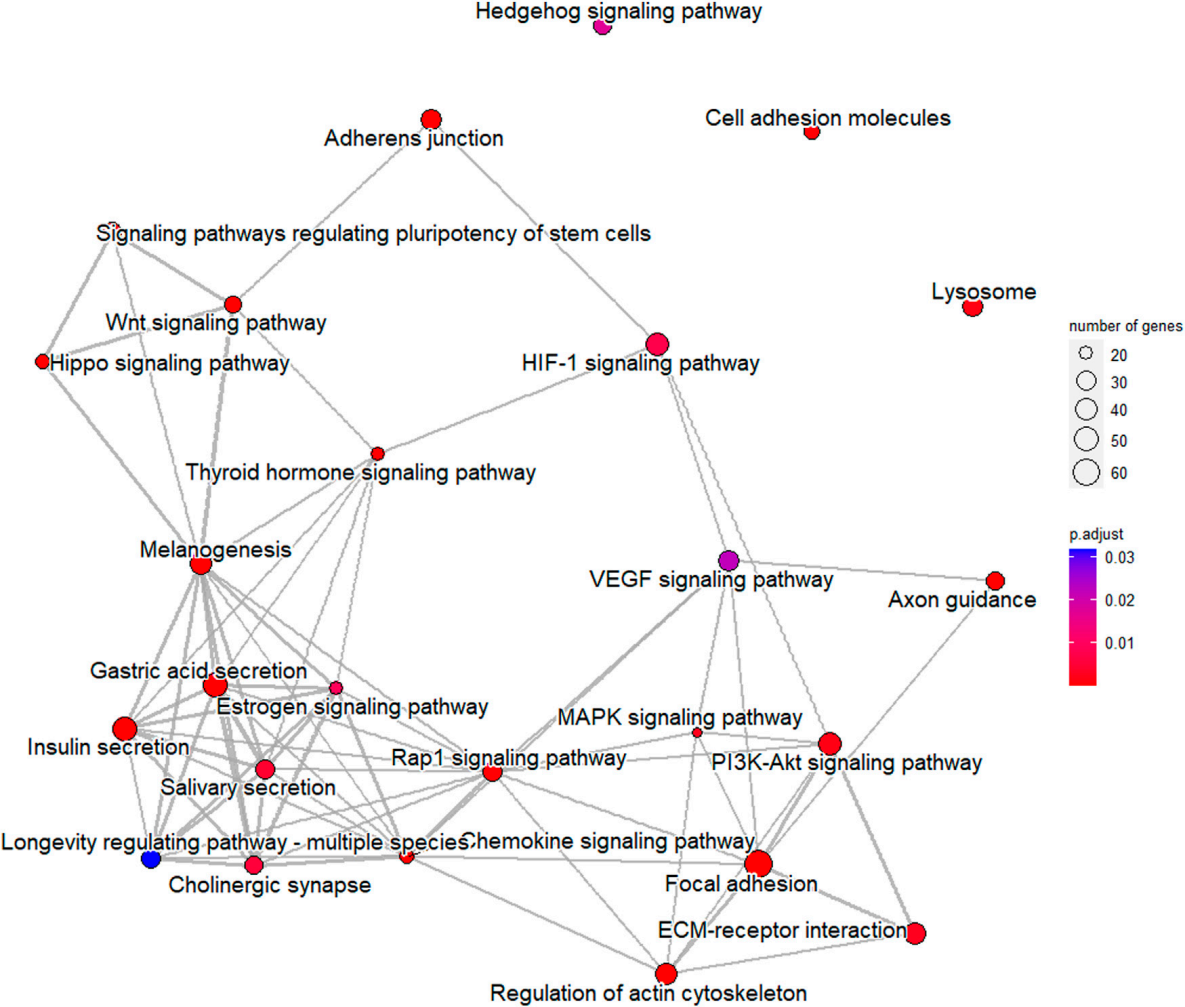
Associations among top 25 significantly enriched pathways for target genes of differentially expressed piRNAs.

**FIGURE 8:**
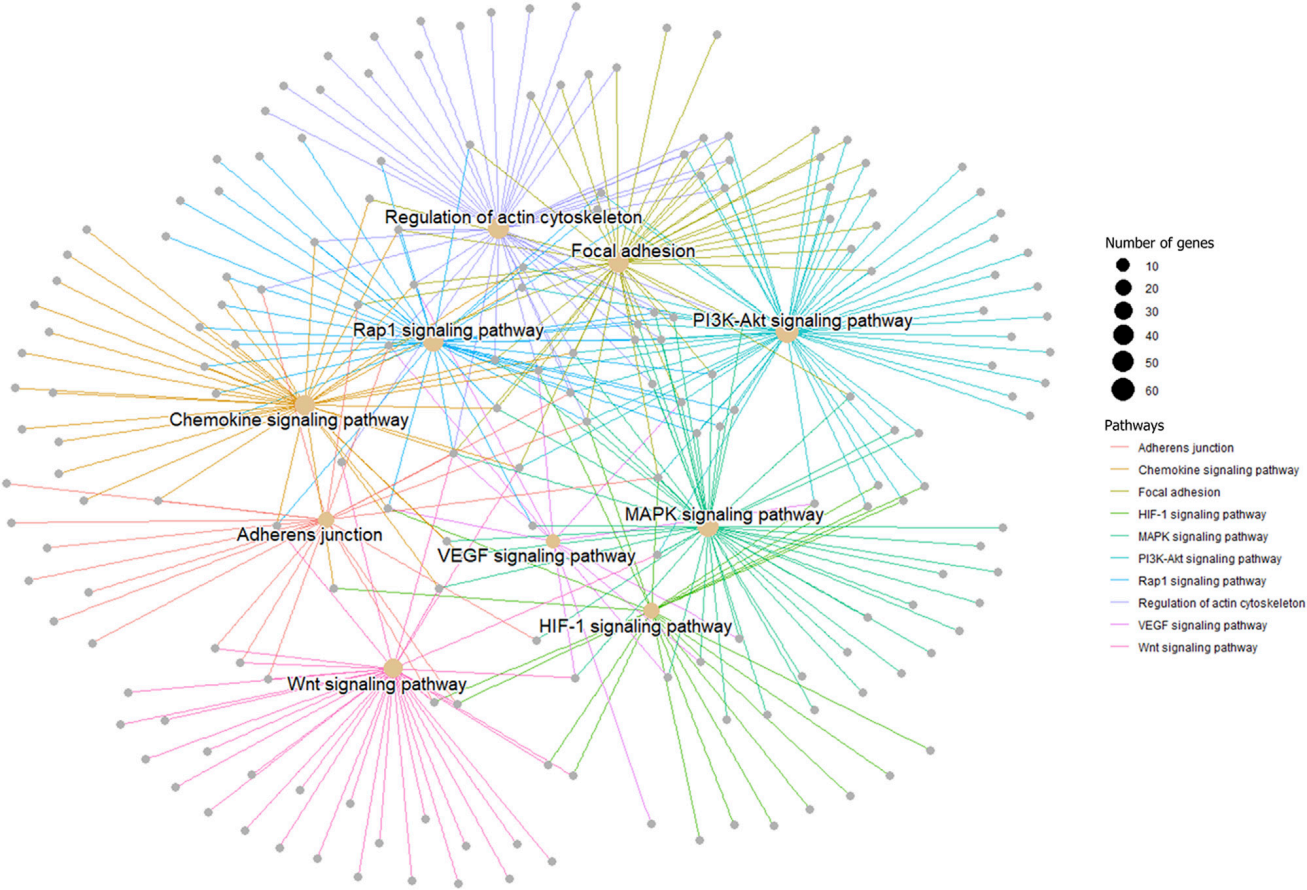
A gene network among male reproduction-related signal pathways. Genes in each pathway are represented by one color.

In order to verify the accuracy of sequencing data, five differentially expressed piRNA sequences were randomly selected for qPCR. The results (Supplementary Figure S1)_showed that the expression trends of five piRNAs were consistent with the sequencing results, indicating that the data obtained from small RNA sequencing were reliable for subsequent analysis. A total of 2,428 DE piRNAs were screened in the testes of rams with different fertility. The top 30 DE piRNAs were summarized in Table 3. The functions of all DE piRNAs were predicted by GO and KEGG enrichment analysis for target genes. In terms of biological processes, their target genes were mainly enriched in Biological regulation, Cellular process, Metabolic process, Response to stimulus, and Single-organism process (Figure 6). In terms of cellular components, they were mainly involved in Cell, Cell part, Membrane and Organelle. In terms of molecular functions, Binding and Catalytic activity were enriched by their target genes. KEGG enrichment analysis showed that the target genes of DE piRNAs were significantly enriched in 54 pathways, and the 25 most significantly pathways were shown in Supplementary Table S2. They included important pathways of reproduction, which were closely related to adhesion (Focal adhesion pathway, Adherens junction, Cell adhesion molecules and Rap1 signaling pathway), spermatogonial stem cell renewal and differentiation (Wnt signaling pathway, PI3K/Akt signaling pathway, MAPK signaling pathway, HIF-1 signaling pathway and Rap1 signaling pathway) and cytoskeleton (Regulation of actin cytoskeleton, Chemokine signaling pathway and Focal adhesion pathway), respectively. Among them, the pathways associated with adhesion and cytoskeleton play important roles in ensuring the stability of the blood-testicular barrier. Notably, cytoscape analysis indicated that the top 25 enriched pathways were closely interconnected (Figure 7). Moreover, genes in these pathways also formed a complex association network (Figure 8), suggesting that they would act synergistically in the regulation of ram reproduction.

**TABLE 3:** Top 30 significantly differentially expressed piRNAs in testes of rams with different fertility.

	piRNA ID	Mean TPM in SNT	Mean TPM in STH	Log2 (Foldchange)	*p* Value
Up-regulated piRNAs in testes of rams with high fertility	t00003531	164.3288	375.8126	1.193428	4.34E-17
	t00003529	162.3703	376.3698	1.212863	4.76E-15
	t00007113	70.77197	218.2707	1.624868	6.52E-13
	t00002797	217.5227	449.2671	1.046407	1.32E-12
	t00009382	63.55267	159.767	1.329945	9.23E-10
	t00003181	156.5043	429.1206	1.45518	4.49E-09
	t00009622	62.8674	154.8359	1.300356	8.74E-09
	t00001771	328.9762	665.1857	1.015774	1.05E-08
	t00007019	93.5032	199.6792	1.094596	1.78E-08
	t00004577	138.9869	291.0943	1.066538	2.83E-08
	t00006386	101.8349	219.3988	1.107,324	3.56E-08
	t00002725	223.7737	457.7331	1.032466	4.14E-08
	t00007398	90.0843	190.3056	1.078971	6.25E-08
	t00007717	81.69437	186.3416	1.189641	7.25E-08
	t00007494	82.5236	193.57	1.229977	1.51E-07
Down-regulated piRNAs in testes of rams with high fertility	t00003368	565.937	0.01	−15.7884	6.08E-54
	t00021600	83.9834	19.8615	−2.08013	2.70E-31
	t00009149	174.2414	56.50243	−1.6247	1.56E-26
	t00018153	91.88513	30.786	−1.57756	2.77E-25
	t00017913	93.2936	30.3104	−1.62197	1.09E-21
	t00049445	42.74047	2.820,867	−3.92139	4.98E-18
	t00101777	21.43303	0.300,867	−6.15457	1.18E-16
	t00002003	601.4204	294.1466	−1.03184	8.47E-16
	t00031563	56.7441	14.91327	−1.92787	1.34E-15
	t00046072	40.0407	9.062367	−2.14351	3.73E-15
	t00001675	777.2703	275.9745	−1.49388	8.28E-15
	t00000321	3258.568	1070.809	−1.60554	8.32E-14
	t00000218	4432.379	1528.403	−1.53606	3.39E-12
	t00039624	43.03687	13.90583	−1.62988	3.51E-12
	t00024428	65.75667	26.38123	−1.31763	1.88E-11

Foldchange: Foldchange value between the two sets of expression values; *p* Value: Probability value of significance test for difference between two sets of expression values.

## 4 Discussion

### 4.1 Characteristics of PIWI-interacting RNA in sheep testes

In this study, the characteristics of piRNA including length distribution, ping-pong signature, strand specificity, genomic origin and chromosomal distribution were revealed in sheep testes. The length distribution of microRNAs (miRNAs) ranges from 18–24 nt (Loganantharaj et al., 2017; Singh and Storey, 2021). However, as a new class of small RNAs, the length range of piRNAs is different from that of microRNAs. In this study, the length of piRNAs reads showed a unimodal distribution between 24 and 33 nt in sheep testes. The length range of piRNAs was similar to those piRNAs in testes of mammals such as mouse (Grivna et al., 2006), horse (Li et al., 2019), yak (Gong et al., 2018) and human (Ha et al., 2014), whereas it was longer than that of piRNAs sequences in reproductive tissues of *Drosophila* (Aravin et al., 2003) and *C. elegans* (Ruby et al., 2006). The piRNA reads and tags in testes and ovaries of mouse also showed a single peak distribution, with the peak appearing at 26 and 27 nt (Yan et al., 2011). The piRNA reads and tags in testes of rhesus macaque also exhibit a single peak distribution, moreover the peak was also consistent with that of sheep (29 nt) (Yan et al., 2011). Our previous study on piRNAs in ovaries showed that peak values of reads number appeared at 24 nt and 27–29 nt, and the number of tags showed a decreasing distribution between 24 and 33 nt (Li et al., 2022). This hinted that sequence distribution of piRNAs has its own characteristics in different tissues of sheep. Studies indicated that different PIWI proteins tend to bind piRNAs with different lengths. For example, Piwi protein tends to bind 25 nt piRNAs while Aub and Ago3 tend to bind 23–24 nt piRNAs in *Drosophila*; MILI tends to bind 28 nt piRNAs while MIWI and MIWI2 tend to bind 30 nt piRNAs in mouse (Aravin et al., 2006; Grivna et al., 2006; Aravin et al., 2008). Our previous study found that four PIWI proteins (MIWI, MILI, MIWI2 and AGO3) were expressed in sheep testes tissue (Li et al., 2020), which was similar with PIWI proteins in mouse. Referring to the study results in mouse, the first three PIWI proteins just bind to 28–30 nt piRNA, which was in good agreement with the peak (28–30 nt) of piRNA reads in this study. Thus, it also implied that the piRNA length and the synergistic PIWI proteins have specificity between mammal and non-mammal.

piRNAs derived from different genomic regions play different biological roles (Aravin et al., 2003; Girard et al., 2006; Yan et al., 2011). Therefore, in this study, alignment regions of the sheep testes-derived piRNA in genome were analyzed. Results showed that the alignment regions of piRNAs were similar in the testes of sheep with different fertility, and they were mainly aligned to the CDS region, repetitive sequence region, intron region and unannotated region. In addition, there were a small number of piRNAs aligned to 5′UTR, 3′UTR and lncRNA. This means that piRNAs in sheep testes also take part in the regulation of gene expression besides inhibiting the activity of transposons. The target region of microRNA includes 3′ UTR, CDS region and 5′ UTR of target mRNA (Liu et al., 2013). Compared with other species, we found that the proportion of piRNAs aligned to repetitive regions (14.98%–15.27%) in sheep testes was similar to that in testes of adult mouse (∼17%) (Grivna et al., 2006) and human (∼22%) (Ha et al., 2014). However, the proportion of piRNA aligned to CDS regions (26.81%–27.64%) in sheep testes was higher than that in mice (∼1.3%) (Grivna et al., 2006) and humans (∼10%) (Ha et al., 2014). This suggested that a larger proportion of piRNAs were involved in gene expression regulation in sheep testes. In addition, the proportion of piRNAs aligned to intronic regions (17.39%–21.35%) was also higher than that of mice (∼9.3%) (Grivna et al., 2006) and pigs (7.6%) (Gebert et al., 2015). In summary, these differences of piRNA distribution in different species suggest that the biological roles of piRNAs were diverse in different species. In addition, it is worth noting that some piRNAs in sheep testes were also aligned to the lncRNA region. Kelley et al. found that lncRNA sequences in human testes tissue contain many retrotransposon sequences (Kelley and Rinn, 2012). It was reported that piRNAs in mouse testes can mediate the degradation of lncRNAs derived from Rasgrf1-marked sites (Watanabe et al., 2011). These results suggest that piRNAs may be also involved in regulating the expression of lncRNAs.

In order to further understand the inhibition of piRNAs for transposons in sheep testes, piRNAs derived from repeat sequences were aligned to the Repbase database to identify the types of transposons. Results showed that piRNAs in sheep testes were aligned predominantly to retrotransposon, which was consistent with the type in human (Ha et al., 2014), pig (Gebert et al., 2015) and yak (Gong et al., 2018). In a finer classification of retrotransposon, piRNAs in humans and pigs mainly aligned to LTR (Ha et al., 2014) and SINE (Gebert et al., 2015) respectively, while sheep and yak (Gong et al., 2018) mainly aligned to LINE type. It suggested that transposon components with high activity, i.e. action targets of piRNA, differs in these species. Furthermore, piRNAs from each TE type (LINE, SINE, and LTR) showed obvious ping-pong cycle characteristics, which implied that ping-pong cycle mechanism was adopted in sheep testes to cleave mRNA produced by transposons in order to inhibit the activity of transposons and maintain the stability of genome.

piRNAs exist in piRNA clusters on chromosomes (Toth et al., 2016; Ozata et al., 2019), which are considered to be the graves of transposons (Brennecke et al., 2007). We identified 284–430 piRNA clusters in each sample of sheep testis, and the numbers are similar with those identified in model animals (Lakshmi and Agrawal, 2008) and bovids (Russell et al., 2017). The first bases of all piRNAs derived from piRNA clusters had a strong U preference, which means that most piRNAs in clusters originated from the primary piRNA pathway. However, this feature does not exist in microRNA sequences. It was previously reported that primary piRNAs in gonadal tissue were generally derived from the antisense strand of transposons and have strong strand specificity (Brennecke et al., 2007; Aravin et al., 2008; Lin and Yin, 2008; Ozata et al., 2019). Therefore, we performed an analysis on the strand specificity of piRNA clusters, and found that within a single piRNA cluster, piRNAs have strong strand specificity. Interestingly, the statistic analysis for all piRNA clusters revealed that the number of piRNAs derived from the sense and antisense strands was comparable and the strand-specificity was no longer obvious. In fact, this phenomenon also exists in pigs (Gebert et al., 2015; Russell et al., 2017), but its specific mechanism need to be further explored.

### 4.2 Prediction of differentially expressed PIWI-interacting RNA function in testes of sheep

Statistical results for indicators related to sperm quality (the ejaculate volume, pH value of semen, sperm viability, sperm motility, sperm density and sperm deformity rate) in SNT and STH rams showed that the single ejaculate volume and sperm number of STH sheep were significantly higher than that of SNT and the pH value of semen was closer to seven in STH sheep. Higher ejaculate volume and more motile sperm should be related to the higher fertility of STH sheep. Bovine sperm motility is highest when the pH of the semen is between 7 and 7.5 (Contri et al., 2013). In this study, the semen pH of STH was closer to seven compared with SNT, implying that sperm from STH had higher motility. All of the above factors contribute to increased fertility in STH rams.

In a succinct summary for signaling pathways enriched by DE piRNA target genes in the testes of rams with different fertility, they were closely related to adhesion, cytoskeleton, spermatogonial stem cell renewal and differentiation, which play crucial roles during spermatogenesis. Studies demonstrated that multiple adhesion proteins could act synergistically to safeguard the function of blood-testis barrier (BTB) (Cheng and Mruk, 2012), that is an important ultrastructure to support spermatogenesis. In this study, four adhesion-related pathways (Focal adhesion pathway, Adherens junction, Cell adhesion molecules and Rap1 signaling pathway) were noted. These pathways affect the normal physiological function of tight junctions of BTB during spermatogenesis (Siu et al., 2009). Loss of tight junctions could result in disruption of the integrity of BTB (Aivatiadou et al., 2007; Le Borgne et al., 2014; Okada et al., 2014), fibrosis of seminiferous tubule epithelial cells (Mazaud-Guittot et al., 2010), apoptosis of germ cell in the meiotic stage, decrease in sperm number and even azoospermia (Wu et al., 2012; Chakraborty et al., 2020). In this study, five pathways related to spermatogonial stem cell renewal and differentiation (Wnt signaling, PI3K/Akt pathway, MAPK signaling pathway, HIF-1 signaling pathway, Rap1 signaling pathway) were also significantly enriched. Previous studies showed that these pathways were involved in the regulation of testicular growth and development (Chambard et al., 2007), specifically playing important roles in proliferation, mitosis, and meiosis of germ cells (Godet et al., 2008; Nicol and Guiguen, 2011; Jiang et al., 2013; Kerr et al., 2014) and proliferation and lactate production of Sertoli cells (Riera et al., 2003; Lucas et al., 2012; Galardo et al., 2013; Yao et al., 2015; Ni et al., 2019) during both early and late spermatogenesis. When the above pathways were disturbed, the proliferation ability of SSCs (Lee et al., 2007; Yao et al., 2020) and Sertoli cell (Niu et al., 2015; Wang et al., 2015; Wang et al., 2017) would be inhibited. In addition, a study on asthenozoospermia population indicated that differentially expressed miRNAs in seminal plasma samples between patients with asthenospermia and healthy men were significantly associated with PI3K-Akt signaling pathway, MAPK signaling pathway and HIF-1 signaling pathway (Liang et al., 2022), which were consistent with our findings. Apart from that, the target genes of DE piRNAs were also significantly enriched in three pathways related to the cytoskeleton: Regulation of actin cytoskeleton, Chemokine signaling pathway and Focal adhesion pathway. Cytoskeletal components in the BTB are critical to development of germ cells along the Sertoli cells and successful spermatogenesis (Li et al., 2018; Wen et al., 2018).

Spermatogenesis is a complex process that is coordinated by multiple pathways. Notably, some of the pathways mentioned above were interrelated. We mapped the network relationships among the top 25 significantly enriched pathways and found that the complex associations exist among pathways (Figure 7). Furthermore, genes in pathways related to male reproduction also formed an associative network with each other (Figure 8). In fact, many previous studies suggested links among these pathways. For example, after VEGF activates the PI3K/Akt signaling pathway, expression of translation regulators (p70S6K and 4EBP1) will raise, and the synthesis of HIF-1α will be promoted (Dery et al., 2005; Marhold et al., 2015; Park et al., 2015; Zhu et al., 2018), which revealed the upstream-downstream relationship among VEGF signaling pathway, PI3K/Akt signaling pathway and HIF-1 signaling pathway. In this study, combined with the results of semen characterization and functional cluster analysis for DE piRNAs, it was implied that the differentially expressed piRNAs in the testes of rams with different fertility might be involved in regulation of BTB stability, proliferation of germ cells and accuracy of the genome through the above-mentioned pathways, which work synergistically to ultimately affect spermatogenesis and sperm number.

## 5 Conclusion

In this study, the sequence characteristics, origin and expression patterns of piRNAs were revealed in sheep testes. The length of piRNAs showed a unimodal distribution between 26 and 31 nt in sheep testes, with a peak at 29 nt. These piRNAs exhibited obvious ping-pong signature and strand specificity. In the genome, they were mainly aligned to CDS, intron, repetitive sequence regions and unannotated regions. Furthermore, in transposon analysis, piRNAs were aligned predominantly to LINE, SINE, and LTR types of retrotransposon in sheep testes, and the piRNAs derived from each type showed obvious ping-pong signature. The piRNA clusters identified in sheep testes were mainly distributed on chromosomes 3, 7, 15, 17, 18 and 20. The results combining semen determination with pathway enrichment analysis implied that differentially expressed piRNAs between the testes of rams with different fertility may participate in spermatogenesis by regulating multiple pathways closely related to stabilization of blood-testis barrier and renewal and differentiation of spermatogonial stem cell. Together, the study provide new insights into the characteristics and expression patterns of piRNAs in sheep testes tissue, which would help us better understand the role of piRNAs in sheep reproduction.

## Data Availability

The datasets presented in this study can be found in online repositories. The names of the repository/repositories and accession number(s) can be found in the article/Supplementary Material

## Supplementary material

Supplementary Figure S1 Results of RNA-seq and qPCR for five random selected piRNAs in testes of SNT and STH sheep.
