# Supplementary material for: Characteristics of piRNAs and their comparative profiling in testes of sheep with different fertility
Source: Front Genet. 2022 Dec 7;13:1078049. doi: 10.3389/fgene.2022.1078049 (PMC9768229; doi:10.3389/fgene.2022.1078049)
Supplement: Supplementary file 1 [file Table1.DOCX]

Table S1 Detailed information for reverse transcription stem-loop primers and qPCR amplification primers

| piRNAs | Sequences of stem-loop primers and qPCR amplification primers |
| --- | --- |
| t00034830 | RT: CTCAACTGGTGTCGTGGAGTCGGCAATTCAGTTGAGGGAGGTTT |
|  | F: GCTGCAAAATCATCAGACTCAGC |
|  | R: CTCAACTGGTGTCGTGGAGTC |
| t00019177 | RT: CTCAACTGGTGTCGTGGAGTCGGCAATTCAGTTGAGGTCACGCT |
|  | F: GCGCCTAATTTGATTTACAGTGTGT |
|  | R: CTCAACTGGTGTCGTGGAGTC |
| t00109064 | RT: CTCAACTGGTGTCGTGGAGTCGGCAATTCAGTTGAGGACAATAT |
|  | F: GCGTCTTGACTCTGGATTAGGAG |
|  | R: CTCAACTGGTGTCGTGGAGTC |
| t00065492 | RT: CTCAACTGGTGTCGTGGAGTCGGCAATTCAGTTGAGCAAGGAAC |
|  | F: GCTAATTTGGGGGAAAGAAAGGC |
|  | R: CTCAACTGGTGTCGTGGAGTC |
| t00009700 | RT: CTCAACTGGTGTCGTGGAGTCGGCAATTCAGTTGAGCATGTAAA |
|  | F: GCGCTATATTTCAAGATACTTGTGGA |
|  | R: CTCAACTGGTGTCGTGGAGTC |
| U6 | RT: AACGCTTCACGAATTTGCGT |
|  | F: GCTTCGGCAGCACATATACT |
|  | R: TTCACGAATTTGCGTGTCAT |
