# Supplementary material for: Characteristics of piRNAs and their comparative profiling in testes of sheep with different fertility
Source: Front Genet. 2022 Dec 7;13:1078049. doi: 10.3389/fgene.2022.1078049 (PMC9768229; doi:10.3389/fgene.2022.1078049)
Supplement: Supplementary file 3 [file Table2.DOCX]

**TABLE S2: Top 25 most significantly enriched pathways for target genes of DE piRNAs**

| **KEGG_A_class** | **KEGG_B_class** | **Pathway** | **P value** | **Q value** | **Pathway ID** |
| --- | --- | --- | --- | --- | --- |
| Organismal Systems | Endocrine system | Insulin secretion | 1.0E-06 | 0.0002 | ko04911 |
| Organismal Systems | Endocrine system | Melanogenesis | 2.0E-06 | 0.0002 | ko04916 |
| Organismal Systems | Development | Axon guidance | 4.0E-06 | 0.0003 | ko04360 |
| Environmental Information Processing | Signaling molecules and interaction | ECM-receptor interaction | 1.1E-05 | 0.0005 | ko04512 |
| ganismal Systems | Endocrine system | Thyroid hormone signaling pathway | 1.6E-05 | 0.0006 | ko04919 |
| Cellular Processes | Cellular community - eukaryotes | Focal adhesion | 3.6E-05 | 0.0012 | ko04510 |
| Cellular Processes | Cellular community - eukaryotes | Adherens junction | 5.2E-05 | 0.0015 | ko04520 |
| Cellular Processes | Cellular community - eukaryotes | Signaling pathways regulating pluripotency of stem cells | 6.8E-05 | 0.0017 | ko04550 |
| Organismal Systems | Immune system | Chemokine signaling pathway | 1.3E-04 | 0.0027 | ko04062 |
| Organismal Systems | Digestive system | Gastric acid secretion | 1.4E-04 | 0.0027 | ko04971 |
| Environmental Information Processing | Signal transduction | HIF-1 signaling pathway | 3.4E-04 | 0.0061 | ko04066 |
| Environmental Information Processing | Signaling molecules and interaction | Cell adhesion molecules (CAMs) | 3.7E-04 | 0.0061 | ko04514 |
| Environmental Information Processing | Signal transduction | Wnt signaling pathway | 4.4E-04 | 0.0067 | ko04310 |
| Organismal Systems | Digestive system | Salivary secretion | 5.1E-04 | 0.0068 | ko04970 |
| Environmental Information Processing | Signal transduction | Hippo signaling pathway | 5.2E-04 | 0.0068 | ko04390 |
| Environmental Information Processing | Signal transduction | PI3K-Akt signaling pathway | 5.7E-04 | 0.0070 | ko04151 |
| Cellular Processes | Cell motility | Regulation of actin cytoskeleton | 8.2E-04 | 0.0092 | ko04810 |
| Cellular Processes | Transport and catabolism | Lysosome | 8.5E-04 | 0.0092 | ko04142 |
| Environmental Information Processing | Signal transduction | Rap1 signaling pathway | 1.1E-03 | 0.0105 | ko04015 |
| Organismal Systems | Endocrine system | Estrogen signaling pathway | 1.7E-03 | 0.0133 | ko04915 |
| Organismal Systems | Nervous system | Cholinergic synapse | 2.0E-03 | 0.0153 | ko04725 |
| Environmental Information Processing | Signal transduction | MAPK signaling pathway | 2.1E-03 | 0.0162 | ko04010 |
| Environmental Information Processing | Signal transduction | Hedgehog signaling pathway | 2.8E-03 | 0.0165 | ko04340 |
| Environmental Information Processing | Signal transduction | VEGF signaling pathway | 7.1E-03 | 0.0235 | ko04370 |
| Organismal Systems | Aging | Longevity regulating pathway - multiple species | 3.1E-03 | 0.0345 | ko04213 |
